# Starving

**Published:** 1890-10-11

**Authors:** 


### THERAPEUTICS.

### STARVING.

Dr. George Shearer, of Liverpool, has some remarks in the last number of the *Provincial Medical Journal*, headed "On Starving into Health," which are well worth attention. He thinks it is a pity that the cases at the Westminster Aquarium were not more carefully and scientifically watched. But sufficient has been proved to raise a question, how this demonstrated capacity of the human body of sustaining fast can be turned to account in the treatment of disease. Cases sometimes occur which appear to show the applicability of treatment by fasting. "Every physician has occasionally met with instances of so-called serous apoplexy, when after a prolonged condition of comatose insensibility, and abstinence of food for many days, the patient at length awakes, to consciousness and health." During the process of unconscious starvation, the effused fluids have been absorbed with a great deal of the bodily substance. A case was recently reported by Dr. Murray, of Newcastle-on-Tyne, where absolute starvation went on for 14 days in an obese subject attacked by serous apoplexy, the termination being recovery, and loss of weight. From this and other experience, Dr. Shearer opines that "it would seem that cases of apoplexy, cerebral congestion, and all kinds of brain pressure, cases of serous effusion into the ventricles of the brain, the cavities of the chest and abdomen, cases of obesity and anasarca, prolific and fibroid tumours, aneurism, gout, alcoholic derangements of the liver and stomach, and many forms of dyspepsia might be benefited if treated by a more or less strict fasting regimen." Although, perhaps, all that Dr. Shearer says may not be fully endorsed, still, there is little doubt that abstinence may come to play a more important part in therapeutics than has yet been accorded to it. In many cases of disease the lightest nourishment is rejected, as if nature would indicate the method of cure by total abstinence from food, and rest for suffering organs. Various forms of dyspepsia, chronic vomiting, ulcer of the stomach, gastric and intestinal catarrh are maladies in which only nominal supplies of food are retained. The physician, however, who designs to make his patients fast, or who even limits the food to a minimum, very often finds his efforts negatived by the obstinacy of relatives. There are a large class of people whose one great idea in illness is to "keep up the patient's strength." And to promote this they will insist on the unfortunate sick swallowing material which they are altogether incapable of digesting. Especially is injury often done by the incautious giving of aliment during the convalescent stages of enteric fever. As the rule, it may be safely stated that the sick are overfed. Also that many of the articles which are given to the sick have little real nutriment value. Even recently our faith in beef-tea has been shaken. It is no less certain that, as a rule, the healthy individual is over-fed. A course of too much eating, especially if combined with a sedentary life, usually terminates in one or other of various diseased conditions. Then colchicum, blue pill, saline purgatives, or other remedies of more recent date and names are employed. Mr. Shearer thinks there is every reason to believe that the same good results could be obtained by bodily rest in a suitable temperature, and fasting. With this we fully agree, though to the average Englishman, and those devoted to the pleasures of the table, this will not appear the more excellent way of cure.

In connection with the above we notice that Dr. Donkin, physician to the Westminster Hospital, advises in the *Lancet*, September 27th, the treatment of gastric ulcer by enemata. The diagnosis of gastric ulcer is difficult, but Dr. Donkin feels sure that it is always wise to recognize, in every protracted case of localized and unerrant pain after food continuing for any length of time, and especially when followed and relieved by vomiting, the possibility of gastric ulcer. Hæmatemesis, as a rule, clinches the diagnosis. Dr. Donkin feels assured that the avoidance of all food given by the mouth, thus keeping the stomach empty and restful, is much the shortest way of relieving and, in some cases, curing gastric ulcers. Small enemata of milk and beef tea should be given at frequent intervals. A report of ten cases is added which all did well under this treatment by enemata. Dr. Donkin does not advise peptonised enemata. He remarks that all the patients waste more or less at first. Dr. Donkin confesses to physiological doubt, whether any albuminous substances are assimilated after rectal feeding, but he will not discuss the question whether the water of the enemata may not be the most valuable element. It has now been amply demonstrated that man can live for many days on water alone. Dr. Donkin adds that in instances when the diagnosis may not be clear, he employs the same method if there is the slightest discomfort produced by oral ingestion of milk or beef tea. Many a case of so-called chronic dyspepsia has been successfully treated in this manner.

